# Ill-Posed Point Neuron Models

**DOI:** 10.1186/s13408-016-0039-8

**Published:** 2016-04-30

**Authors:** Bjørn Fredrik Nielsen, John Wyller

**Affiliations:** Department of Mathematical Sciences and Technology, Norwegian University of Life Sciences, P.O. Box 5003, Ås, 1432 Norway

**Keywords:** Point-neuron models, Ill posed, Numerical solution

## Abstract

We show that point-neuron models with a Heaviside firing rate function can be ill posed. More specifically, the *initial-condition*-to-*solution* map might become discontinuous in finite time. Consequently, if finite precision arithmetic is used, then it is virtually impossible to guarantee the accurate numerical solution of such models. If a smooth firing rate function is employed, then standard ODE theory implies that point-neuron models are well posed. Nevertheless, in the steep firing rate regime, the problem may become close to ill posed, and the error amplification, in finite time, can be very large. This observation is illuminated by numerical experiments. We conclude that, if a steep firing rate function is employed, then minor round-off errors can have a devastating effect on simulations, unless proper error-control schemes are used.

## Introduction

Modeling of electrical potentials has a long tradition in computational neuroscience. One model with some physiological significance is the voltage-based system

1 $$\begin{aligned} \boldsymbol {\tau} \mathbf{u}'(t) &= - \mathbf{u}(t) + \boldsymbol {\omega} S_{\beta}\bigl[\mathbf{u}(t)- \mathbf{u}_{\theta}\bigr] + \mathbf{q}(t), \quad t \in(0,T], \end{aligned}$$

2 $$\begin{aligned} \mathbf{u}(0)&=\mathbf{u}_{0}, \end{aligned}$$

where

$$\begin{aligned} &\mathbf{u}(t), \mathbf{q}(t) \in \mathbb {R}^{N},\quad t \in(0,T], \\ &\mathbf{u}_{\theta}, \mathbf{u}_{0} \in \mathbb {R}^{N}, \\ &\boldsymbol {\omega} \in \mathbb {R}^{N \times N}, \\ &\boldsymbol {\tau} \in \mathbb {R}^{N \times N} \mbox{ is diagonal}, \\ &S(x) = \frac{1}{2}\bigl(1+\tanh(x)\bigr), \\ &S_{\beta}[x] = S(\beta x), \\ &S_{\beta}[\mathbf{x}]=\bigl(S_{\beta}[x_{1}], \ldots,S_{\beta}[x_{N}]\bigr)^{T}, \quad\mathbf{x} = (x_{1}, \ldots, x_{N})^{T} \in \mathbb {R}^{N}. \end{aligned}$$

In the rate model (1)–(2), each component function $u_{i}(t)$ of $\mathbf{u}(t)$ represents the time dependent potential of the *i*th unit in a network of *N* units. The nonlinear function $S_{\beta}$ is called the firing rate function, $\{ \omega_{ij} \}$ are the connectivities, and $\mathbf{q}(t)$ models the external drive. A detailed derivation of this model can be found in [1–3].

The purpose of this paper is to explore the properties of the *initial-condition*-to-*solution* map

3 $$ R_{\beta}: \mathbf{u}_{0} \rightarrow \mathbf{u}(T),\quad T < \infty, $$

associated with (1)–(2). Note that we use the subscript *β* to emphasize that $R_{\beta}$ depends on the steepness parameter *β*, and that $R_{\infty}$ corresponds to using a Heaviside firing rate function, i.e. $S_{\infty} = H$. We will also make use of the standard notation

4 $$ \|\mathbf{f}\|_{\infty}=\sup_{1\leq i\leq N}|f_{i}|,\quad \mathbf{f}=(f_{1},\ldots,f_{N}), $$

for the supremum norm throughout this paper.

A simple example, presented in Sect. 4, shows that $R_{\infty}$ can become discontinuous. Hence, the model is mathematically ill posed [4, 5] and round-off errors of any size can corrupt computations. We conclude that it is very difficult to produce reliable simulations with such models. Since all norms for finite dimensional spaces are equivalent, it is not possible to “circumvent” this problem by changing the involved topologies.

According to standard ODE theory (Appendix A), $R_{\beta}$, with $\beta< \infty$, is continuous, but the size of the error-amplification ratio

5 $$ E(T;\beta)= \frac{\| \mathbf {u}(T;\beta) - \tilde{\mathbf{u}}(T;\beta) \|_{\infty }}{\| \mathbf{u}_{0} - \tilde{\mathbf{u}}_{0} \|_{\infty}} $$

may be huge for large *β*, which will be demonstrated and analyzed in Sects. 2 and 3, respectively. Here, $\tilde{\mathbf{u}}_{0}$ represents a perturbed initial condition and $\tilde{\mathbf{u}}(t)$ its associated solution. This implies that, also for $1 \ll\beta< \infty$, it can become difficult to guarantee the accurate numerical solution of (1)–(2): Minor round-off errors may be significantly amplified within short time intervals, which can lead to erroneous simulations.

Our investigation is motivated by the fact that steep sigmoid functions, or even the Heaviside function, often are employed in mathematical/computational neuroscience; see e.g. [1, 6] and references therein. Other authors [7, 8] have also pointed out that severe challenges occur if $\beta= \infty$, i.e. issues concerning how to define suitable function spaces and to prove existence of solutions. Nevertheless, as far as we know, results which explicitly discuss the ill-posed nature of (1)–(2) when $\beta=\infty$, and how this property yields extra numerical challenges in the steep, but smooth, firing rate regime, has not previously been published.

### Remark

We would like to point-out the following: Assume that an initial condition is close to an unstable equilibrium. Our results should not be interpreted as expressing the mundane fact that a perturbation of this initial condition, moving it to another region with completely different dynamical properties, may lead to large changes in the solution. In fact, we show that the error-amplification ratio can be huge, during small time intervals, even though the perturbation does not change which neurons are active. That is, the change in the initial condition is not such that it changes the qualitative behavior of the dynamical system for $0< t \ll 1$—only the quantitative properties are dramatically altered. This can happen in the steep firing rate regime.

## Numerical Results

Let us first compute the error-amplification ratio (5) for some simple problems.

### Example 1

Consider the following model of a single point neuron, i.e. $N=1$,

$$\begin{aligned} u'(t) =& -u(t)+0.9 S_{\beta} \bigl[u(t)-0.6\bigr]+0.151,\quad t \in(0,T], \\ u(0) =& u_{0} = 0.6. \end{aligned}$$

We used Matlab’s ode45 solver, with the default error-control settings and $T=0.1$, to compute numerical approximations of

$$u(t)=u(t; \beta)\quad\mbox{for } \beta=1, \dots, 200. $$

Also a second series of simulations were performed, using the same selection of values for the steepness parameter, but with the perturbed initial condition

6 $$ \tilde{u}_{0} = u_{0} - 10^{-5}. $$

The corresponding solution is denoted $\tilde{u}(t)=\tilde{u}(t; \beta)$.

**Fig. 1:**
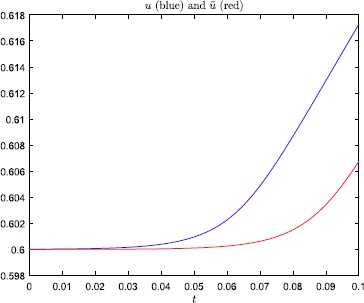
Numerical results, with steepness parameter $\beta=200$, for the problem studied in Example 1

**Fig. 2:**
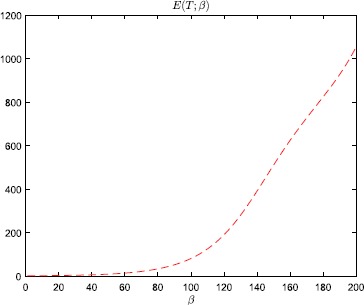
Error-amplification ratio, with $T=0.1$, as a function of the steepness parameter *β* for the problem studied in Example 1

Plots of *u* and *ũ*, with $\beta=200$, are displayed in Fig. 1. Note that in both cases the neuron fires, i.e. the change in the initial condition is not such that it has moved from one side of an unstable equilibrium to the other side. Even so, according to Fig. 2 and Table 1, the error-amplification ratio $E(T;\beta)$, due to the minor perturbation (6) of the initial condition, is in the range $[80.6, 1054.1]$ for $\beta \in[100, 200]$, and also very large for $\beta= 50, 75$.

**Table 1:** **Error-amplification ratio, with** $\pmb{T=0.1}$ **, associated with Example** **1**

*β*	1	25	50	75
$E(T;\beta)=\frac{| u(T;\beta) - \tilde{u}(T;\beta) |}{| u_{0} - \tilde {u}_{0} |}$	0.95	2.79	8.58	26.41

Simulations with the strict error-control setting

$$\mathtt {odeset('RelTol',1e-13,'AbsTol',1e-13)} $$

generated the same results, and so did an explicit Euler scheme, with uniform time-step $\Delta t= 10^{-7}$.

### Example 2

Let us consider a model of two point neurons:

$$\begin{gathered} \begin{aligned}[b] u'_{1}(t) &= -u_{1}(t)+0.9 S_{\beta} \bigl[u_{1}(t)-0.6\bigr]+1.0 S_{\beta} \bigl[u_{2}(t)-0.6 \bigr]-0.3492,\quad t \in(0,T],\\ u'_{2}(t) &= -u_{2}(t)-0.1 S_{\beta} \bigl[u_{1}(t)-0.6\bigr]+0.6 S_{\beta} \bigl[u_{2}(t)-0.6\bigr]+0.3501,\quad t \in(0,T],\\ u_{1}(0) &= u_{1,0} = 0.6,\\ u_{2}(0) &= u_{2,0} = 0.6. \end{aligned} \end{gathered}$$

The same procedure as in Example 1 was used, but with the perturbed initial condition

$$\begin{aligned} \tilde{u}_{1}(0) =& \tilde{u}_{1,0} = u_{1,0} - 10^{-5},\\ \tilde{u}_{2}(0) =& \tilde{u}_{2,0} = u_{2,0} + 10^{-5}. \end{aligned}$$

**Fig. 3:**
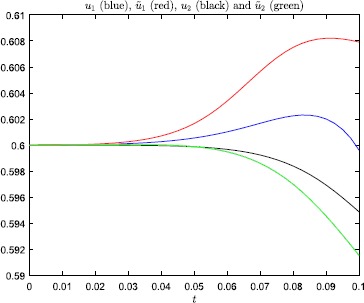
Numerical results, with steepness parameter $\beta=200$ and $T=0.1$, for the problem studied in Example 2

**Fig. 4:**
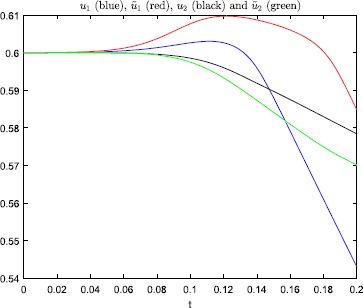
Numerical results, with steepness parameter $\beta=150$ and $T=0.2$, for the problem studied in Example 2

**Fig. 5:**
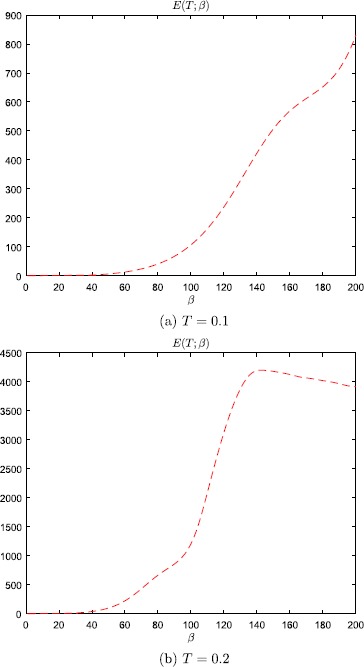
Error-amplification ratio as a function of the steepness parameter *β* for the problem studied in Example 2

Figures 3 and 4 show that this minor change of the initial condition, in the steep firing rate regime, has a huge impact on the solution of the model. And, the perturbation does not change which neuron that fires. In Fig. 5 we have plotted the error-amplification ratio $E(T;\beta)$, see (5), as a function of $\beta=1,2,\ldots,200$. Clearly, in this case $E(T;\beta)$ is unacceptably large, even for rather moderate values of the steepness parameter.

As in Example 1, we used Matlab’s ode45 solver with the standard settings. Computations with the strict error-control parameters

7 $$ \mathtt {odeset('RelTol',1e-13,'AbsTol',[1e-13 1e-13])} $$

produced virtually the same results. The simulations were also “confirmed” by our explicit Euler implementation with time-step $\Delta t = 10^{-7}$.

**Fig. 6:**
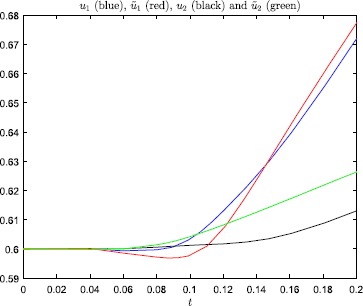
Results generated by Matlab’s ode15s solver, with steepness parameter $\beta=150$ and $T=0.2$. Note that the *curves* are very different from the graphs produced with the ode45 solver; see Fig. 4

Figure 6 shows numerical results computed with Matlab’s ode15s solver, employing the default error-control settings. The curves shown in this figure are very different from the graphs displayed in Fig. 4, which were computed by the ode45 software. We conclude that even the toy example considered in this section is not trivial to solve (with the strict error-control setting (7), ode15s also managed to produce the curves shown in Fig. 4).

**Fig. 7:**
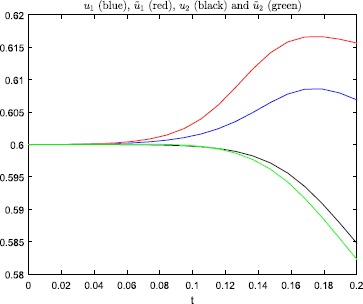
Results generated by an explicit Euler scheme: $\Delta t = 0.01$, $\beta=150$, and $T=0.2$. Note that the *curves* are rather different from the graphs produced with the ode45 solver; see Fig. 4

If $u_{1}(t) \approx u_{\theta}$ and $u_{2}(t) \approx u_{\theta}$, then

$$\bigl|u_{1}(t)\bigr|, \bigl|u_{2}(t)\bigr| \leq2u_{\theta} = 2 \cdot 0.6 = 1.2, $$

and the model implies that

$$\begin{aligned} \bigl|u'_{1}(t)\bigr| \leq& 1.2 + 0.9 + 1.0 + 0.3492 = 3.4492, \\ \bigl|u'_{2}(t)\bigr| \leq& 1.2 +0.1+0.6+0.3501 = 2.2501, \end{aligned}$$

which are rather small. One therefore might think that it is sufficient to employ a moderate time-step to obtain an accurate numerical approximation. Figure 7 shows that this is not the case. (In computational mathematics it is well known that the accuracy of the finite difference approximation $[u_{1}(t+\Delta t) - u_{1}(t)]/\Delta t$, of the derivative $u'_{1}(t)$, depends on the second order derivative $u''_{1}(t)$, which in our case is of order $O(\beta)$. This explains the poor approximation obtained with time-step $\Delta t = 0.01$.)

## Analysis

The purpose of this section is to present an analysis of the error-amplification ratio (5) and thereby explain the main features of our numerical results. Even though the Picard–Lindelöf theorem [9, 10] asserts that (1)–(2) has a unique solution $\mathbf{u}(t)$, provided that $\mathbf{q}(t)$ is continuous and that $\beta< \infty$, it is virtually impossible to determine a simple expression for $\mathbf {u}(t)$. On the other hand, if $\mathbf{u}(t) \approx\mathbf {u}_{\theta }$ and $\beta< \infty$, then we can linearize $S_{\beta}$ to get an approximate model, which is much easier to work with.

### Linearization

The linearization of $S_{\beta}$ about zero reads

8 $$\begin{aligned} L_{\beta} (x) =& S_{\beta} [0] + S'_{\beta} [0] x \\ =& \frac{1}{2} + \frac{1}{2} \beta x. \end{aligned}$$

Define $\boldsymbol {\tau} = \mathbf{I}$, the identity matrix, then the linear approximation of (1)–(2) reads

9 $$\begin{aligned} \mathbf{s}'(t) =& - \mathbf{s}(t) + \boldsymbol {\omega} L_{\beta}\bigl[\mathbf{s}(t)-\mathbf{u}_{\theta}\bigr] + \mathbf{q}(t) \\ =& - \mathbf{s}(t) + \boldsymbol {\omega} \biggl[ \frac{1}{2} \mathbf{1} + \frac{1}{2} \beta\bigl\{ \mathbf{s}(t)-\mathbf{u}_{\theta} \bigr\} \biggr] + \mathbf{q}(t) \\ =& \biggl( \frac{1}{2} \beta\boldsymbol {\omega} - \mathbf{I} \biggr) \mathbf{s}(t) + \frac{1}{2} \boldsymbol {\omega} (\mathbf {1}-\beta \mathbf{u}_{\theta}) + \mathbf{q}(t) \\ =& \mathbf{A} \mathbf{s}(t) + \mathbf{d} + \mathbf{q}(t), \end{aligned}$$

10 $$\begin{aligned} \mathbf{s}(0) =&\mathbf{u}_{0}, \end{aligned}$$

where

11 $$\begin{aligned} \mathbf{A} =& \mathbf{A} (\beta) = \frac{1}{2} \beta \boldsymbol {\omega} - \mathbf{I}, \\ \mathbf{d} =& \mathbf{d}(\beta) = \frac{1}{2} \boldsymbol {\omega} ( \mathbf{1}-\beta\mathbf{u}_{\theta}). \end{aligned}$$

The linearized problem with a perturbed initial condition becomes

$$\begin{aligned} \tilde{\mathbf{s}}'(t) =& \mathbf{A} \tilde{\mathbf{s}}(t) + \mathbf{d} + \mathbf{q}(t), \\ \tilde{\mathbf{s}}(0) =&\tilde{\mathbf{u}}_{0}, \end{aligned}$$

and the difference $\mathbf{s}(t) - \tilde{\mathbf{s}}(t)$ obeys

$$\begin{aligned} \bigl[\mathbf{s}(t) - \tilde{\mathbf{s}}(t)\bigr]' =& \mathbf{A} \bigl[\mathbf{s}(t) - \tilde{\mathbf{s}}(t)\bigr], \\ \mathbf{s}(0) - \tilde{\mathbf{s}}(0) =& \mathbf{u}_{0} - \tilde{ \mathbf{u}}_{0}. \end{aligned}$$

Therefore,

$$\mathbf{s}(t) - \tilde{\mathbf{s}}(t) = (\mathbf{u}_{0} - \tilde{\mathbf{u}}_{0}) e^{\mathbf{A} t}, $$

and the error-amplification ratio can be written in the form

12 $$ \frac{\| \mathbf{s}(T) - \tilde{\mathbf{s}}(T) \|_{\infty}}{\| \mathbf {u}_{0} - \tilde{\mathbf{u}}_{0} \|_{\infty}} =\frac{\| (\mathbf{u}_{0} - \tilde{\mathbf{u}}_{0}) e^{\mathbf{A} T} \| _{\infty}}{\| \mathbf{u}_{0} - \tilde{\mathbf{u}}_{0} \|_{\infty}}. $$

Since the entries of $\mathbf{A} = \mathbf{A} (\beta)$ are of order $O(\beta)$, see (11), we conclude that the error-amplification ratio for the linearized model is of exponential order $O(e^{\beta})$. Is this also the case for the highly nonlinear model (1)–(2)? We will now explore this issue, but first we would like to make a short remark.

#### Remark

Recall the definition (8) of $L_{\beta}$. If we replace $S_{\beta}$ in (1)–(2) with

$$\tilde{L}_{\beta} (x)= \left \{ \textstyle\begin{array}{@{}l@{\quad}l} 1,& x > \frac{1}{\beta}, \\ L_{\beta}(x),& x \in[ -\frac{1}{\beta}, \frac{1}{\beta} ], \\ 0, & x < -\frac{1}{\beta}, \end{array}\displaystyle \right . $$

then the analysis of the linearized model, presented above, would also be valid for (1)–(2), provided that

$$\bigl\| \mathbf{s}(t) - \mathbf{u}_{\theta} \bigr\| _{\infty}, \bigl\| \tilde{ \mathbf{s}}(t) - \mathbf{u}_{\theta} \bigr\| _{\infty} \in\biggl[ 0, \frac{1}{\beta} \biggr], \quad t \in[0,T]. $$

Similarly to the sigmoid function $S_{\beta}$, $\tilde{L}_{\beta}$ also converges point-wise to the Heaviside function as $\beta\rightarrow \infty$. If one employs the sigmoid function in the point-neuron model, then the analysis, as we will see below, becomes much more involved.

### Preparations

Let $\beta_{\max}$, *T̂*, and *α* be arbitrary positive constants. It is easy to construct a smooth vector-valued function **z** satisfying

13 $$ \bigl\| \mathbf{z}(t) - \mathbf{u}_{\theta} \bigr\| _{\infty} \in\biggl[ 0, \frac{0.9}{\beta_{\max}^{1 + \alpha}} \biggr],\quad t \in[0,\hat{T}]. $$

Hence, defining the source as

$$\mathbf{q}(t) = \boldsymbol {\tau} \mathbf{z}'(t) + \mathbf{z}(t) - \boldsymbol {\omega} S_{\beta_{\max}}\bigl[\mathbf{z}(t)-\mathbf{u}_{\theta} \bigr],\quad t \in(0,\hat{T}], $$

we conclude that the solution $\mathbf{u}(t; \beta_{\max}) = \mathbf {z}(t)$ of (1)–(2) also satisfies (13), provided that $\mathbf{u}_{0} = \mathbf{z}(0)$. By employing standard techniques, one can show that the solution $\mathbf{u}(t; \beta)$ of (1)–(2) depends continuously on $0 < \beta< \infty$; see Appendix B. Consequently, there exists $\bar{\beta}_{\min} < \beta_{\max}$ such that

$$\bigl\| \mathbf{u}(t; \beta) - \mathbf{u}_{\theta} \bigr\| _{\infty} \in \biggl[ 0, \frac{1}{\beta_{\max}^{1 + \alpha}} \biggr],\quad t \in[0,\hat{T}], \beta\in[\bar{ \beta}_{\min}, \beta_{\max}]. $$

For the sake of simple notation, we will in our analysis write **u**, or $\mathbf{u}(t)$, instead of $\mathbf{u}(t; \beta)$.

Furthermore, according to the analysis presented in Appendices A–C, *u* depends continuously on both the initial condition $\mathbf{u}_{0}$ and the steepness parameter *β*, when $0 < \beta< \infty$. Motivated by this property of (1)–(2), we assume that both **u** and $\tilde{\mathbf{u}}$, where $\tilde{\mathbf{u}}$ denotes the solution of (1) generated by a perturbed initial condition $\tilde{\mathbf{u}}(0)=\tilde{\mathbf{u}}_{0}$, satisfy

14 $$ \bigl\| \mathbf{u}(t) - \mathbf{u}_{\theta} \bigr\| _{\infty}, \bigl\| \tilde{\mathbf{u}}(t) - \mathbf{u}_{\theta} \bigr\| _{\infty} \in\biggl[ 0, \frac{1}{\beta_{\max}^{1 + \alpha}} \biggr],\quad t \in[0,\hat {T}], \beta\in[\hat{ \beta}_{\min}, \beta_{\max}], $$

where $\hat{\beta}_{\min} < \beta_{\max}$. Then, by invoking the triangle inequality, we find that

15 $$ \bigl\| \mathbf{u}(t) - \tilde{\mathbf{u}}(t) \bigr\| _{\infty} \leq \frac{2}{\beta_{\max}^{1 + \alpha}}, \quad t \in[0,\hat{T}], \beta\in [\hat{\beta}_{\min}, \beta_{\max}], $$

which will be small if $\beta_{\max}$ is large. Even so, as will become evident below, the error-amplification ratio (5) can be significant and lead to erroneous results.

Let **s** and $\tilde{\mathbf{s}}$ denote the associated solutions of the linearized model (9)–(10). From (14) we find that the initial conditions $\mathbf {u}_{0}$ and $\tilde{\mathbf{u}}_{0}$ satisfy

$$\| \mathbf{u}_{0} - \mathbf{u}_{\theta} \|_{\infty}, \| \tilde{\mathbf{u}}_{0} - \mathbf{u}_{\theta} \|_{\infty} \in\biggl[ 0, \frac{1}{\beta_{\max}^{1 + \alpha}} \biggr]. $$

Since **s** and $\tilde{\mathbf{s}}$ are continuous with respect to *t*, the same initial conditions are employed in the linearized model, and these solutions depend continuously on $0< \beta< \infty$, it follows that there exist $\tilde{T} > 0$ and $\tilde{\beta}_{\min} < \beta_{\max}$ such that

16 $$ \bigl\| \mathbf{s}(t) - \mathbf{u}_{\theta} \bigr\| _{\infty}, \bigl\| \tilde{\mathbf{s}}(t) - \mathbf{u}_{\theta} \bigr\| _{\infty} \in\biggl[ 0, \frac{1}{\beta_{\max}^{1 + \alpha}} \biggr],\quad t \in [0,\tilde{T}], \beta\in[\tilde{ \beta}_{\min}, \beta_{\max}]. $$

The main point of this discussion is to show that there exist (smooth) source terms **q** and perturbations of the initial condition such that (14) holds, regardless how large $\hat{T},\beta_{\max},\alpha> 0$ are. Also, the solutions of the linearized model will satisfy (16). For the sake of simple notation, let $T=\min\{ \tilde{T},\hat{T} \}$ and $\beta_{\min} = \max\{ \tilde{\beta}_{\min}, \hat{\beta}_{\min } \}$.

The triangle inequality implies that

$$\begin{aligned} \mathbf{e}(t) =& \mathbf{u}(t)-\mathbf{s}(t), \\ \tilde{\mathbf{e}}(t) =& \tilde{\mathbf{u}}(t)-\tilde{\mathbf{s}}(t) \end{aligned}$$

obey

17 $$ \bigl\| \mathbf{e}(t) \bigr\| _{\infty}, \bigl\| \tilde{\mathbf{e}}(t) \bigr\| _{\infty} \in\biggl[ 0, \frac{2}{\beta_{\max}^{1 + \alpha }} \biggr], \quad t \in[0,T], \beta\in[ \beta_{\min}, \beta_{\max}]. $$

We will derive a bound for $\| \mathbf{e}(T) \|_{\infty}$. The analysis of $\| \tilde{\mathbf{e}}(T) \|_{\infty}$ is completely analogous, and thus it is omitted.

### Linearization Error

Subtracting (9) from (1), and keeping in mind that we consider the case $\boldsymbol {\tau} = \mathbf {I}$, yields

$$e'_{i}(t)= -e_{i}(t)+\sum _{j} \omega_{i,j} \bigl[ S_{\beta} \bigl(u_{j}(t)-u_{\theta }\bigr) - L_{\beta} \bigl(s_{j}(t)-u_{\theta}\bigr) \bigr], $$

$i=1,2,\ldots,N$, where we use the notation $\mathbf{e}(t) = [e_{1}(t), e_{2}(t), \ldots, e_{N}(t)]^{T}$, and similarly for the entries of $\mathbf{u}(t)$ and $\mathbf{s}(t)$. Integrating and invoking the fact that $e_{i}(0) = 0$, we get

18 $$ e_{i}(T)= - \int_{0}^{T} e_{i}(t)\,dt + \int_{0}^{T} \sum_{j} \omega_{i,j} \bigl[ S_{\beta} \bigl(u_{j}(t)-u_{\theta} \bigr) - L_{\beta} \bigl(s_{j}(t)-u_{\theta}\bigr) \bigr]\,dt, $$

$i=1,2,\ldots,N$.

The triangle inequality, Taylor’s theorem and Eq. (8) for $L_{\beta}$ imply that

$$\begin{aligned} \bigl|S_{\beta} \bigl(u_{j}(t)-u_{\theta}\bigr) - L_{\beta} \bigl(s_{j}(t)-u_{\theta}\bigr)\bigr| \leq& \bigl|S_{\beta} \bigl(u_{j}(t)-u_{\theta}\bigr) - L_{\beta} \bigl(u_{j}(t)-u_{\theta}\bigr)\bigr| \\ &{}+ \bigl|L_{\beta} \bigl(u_{j}(t)-u_{\theta}\bigr) - L_{\beta} \bigl(s_{j}(t)-u_{\theta}\bigr)\bigr| \\ \leq& \beta^{2} \frac{1}{2} \max _{y} \bigl|S''(y)\bigr| \bigl(u_{j}(t)-u_{\theta} \bigr)^{2} \\ &{}+ \frac{1}{2} \beta\bigl| e_{j} (t)\bigr| \\ \leq& \beta^{2} \beta^{-2-2\alpha} \\ &{}+ \frac{1}{2} \beta\bigl| e_{j} (t)\bigr| \\ \leq& \beta^{-2\alpha} + \frac{1}{2} \beta\bigl| e_{j} (t)\bigr|, \end{aligned}$$

where the second last inequality follows from (14). By combining this with (18), and the triangle inequality, one finds that

$$\begin{aligned} \bigl|e_{i}(T)\bigr| \leq& \int_{0}^{T} \bigl|e_{i}(t)\bigr|\,dt + B T \beta^{-2\alpha} + \frac{1}{2} \beta B \int_{0}^{T} \bigl\| \mathbf{e}(t) \bigr\| _{\infty}\,dt \\ \leq& \int_{0}^{T} \biggl( 1+\frac{1}{2} B \beta \biggr) \bigl\| \mathbf{e}(t) \bigr\| _{\infty}\,dt + B T \beta ^{-2\alpha}, \end{aligned}$$

where

$$B=\max_{i} \sum_{j} | \omega_{i,j}|. $$

Since this must hold for $i=1,2,\ldots,N$,

$$\bigl\| \mathbf{e}(T) \bigr\| _{\infty} \leq \int_{0}^{T} \biggl( 1+\frac{1}{2} B \beta \biggr) \bigl\| \mathbf{e}(t) \bigr\| _{\infty}\,dt + B T \beta ^{-2\alpha}, $$

and Grönwall’s inequality implies that

19 $$ \bigl\| \mathbf{e}(T) \bigr\| _{\infty} \leq B T \beta^{-2\alpha} \exp\biggl[ \biggl( 1+\frac{1}{2} B \beta\biggr)T \biggr]. $$

### Error-Amplification Ratio

Clearly,

20 $$\begin{aligned} \mathbf{u} - \tilde{\mathbf{u}} =& \mathbf{u} - \mathbf{s} + \mathbf{s} - \tilde{\mathbf{s}} + \tilde{\mathbf{s}} - \tilde{\mathbf{u}} \\ =& \mathbf{e} + \mathbf{s} - \tilde{\mathbf{s}} - \tilde{\mathbf{e}}, \end{aligned}$$

and the reverse triangle inequality yields

$$\| \mathbf{u} - \tilde{\mathbf{u}} \|_{\infty} \geq\bigl\vert \| \mathbf{s} - \tilde{\mathbf{s}} \|_{\infty} - \| \mathbf{e} - \tilde{\mathbf{e}} \|_{\infty} \bigr\vert . $$

From (12) it follows that the error-amplification ratio (5) satisfies

$$\begin{aligned} E(T;\beta) =& \frac{\| \mathbf{u}(T;\beta) - \tilde{\mathbf {u}}(T;\beta) \|_{\infty }}{\| \mathbf{u}_{0} - \tilde{\mathbf{u}}_{0} \|_{\infty}} \\ \geq& \biggl\vert \underbrace{\frac{\| (\mathbf{u}_{0} - \tilde {\mathbf{u}}_{0}) e^{\mathbf{A} T} \|_{\infty}}{\| \mathbf{u}_{0} - \tilde{\mathbf {u}}_{0} \| _{\infty}}}_{I=I(T;\beta)} - \underbrace{\frac{\| \mathbf{e}(T) - \tilde{\mathbf{e}}(T) \| _{\infty }}{\| \mathbf{u}_{0} - \tilde{\mathbf{u}}_{0} \|_{\infty }}}_{\textit{II}=\textit{II}(T;\beta)} \biggr\vert . \end{aligned}$$

Recall that the entries of the matrix $A=A(\beta)$ are of order *β*; see (11). To derive a bound for $\textit{II}(T;\beta)$, we employ (19), and a similar inequality for $\|\tilde{\mathbf{e}}(T) \|_{\infty}$,

$$\begin{aligned} \frac{\| \mathbf{e}(T) - \tilde{\mathbf{e}}(T) \|_{\infty}}{\| \mathbf{u}_{0} - \tilde{\mathbf{u}}_{0} \|_{\infty}} \leq& \frac{2B T \beta ^{-2\alpha} \exp[ ( 1+\frac{1}{2} B \beta )T ]}{\| \mathbf{u}_{0} - \tilde{\mathbf{u}}_{0} \|_{\infty}} \\ =& \beta^{-2\alpha} \frac{2B T}{\| \mathbf{u}_{0} - \tilde{\mathbf{u}}_{0} \|_{\infty}} \exp\biggl[ \biggl( 1+ \frac{1}{2} B \beta\biggr)T \biggr]. \end{aligned}$$

Hence, if

21 $$ \frac{2B T}{\| \mathbf{u}_{0} - \tilde {\mathbf{u}}_{0} \|_{\infty}} $$

is not very large, *β* is fairly large and, e.g., $\alpha\geq 0.5$, then the size of the error-amplification ratio $E(T;\beta)$ is dominated by $I(T;\beta)$, i.e. by the term stemming from the linearized model. (Note that (20) and the reverse triangle inequality also imply that $|E(T;\beta)-I(T;\beta)| \leq \textit{II}(T;\beta)$.)

**Fig. 8:**
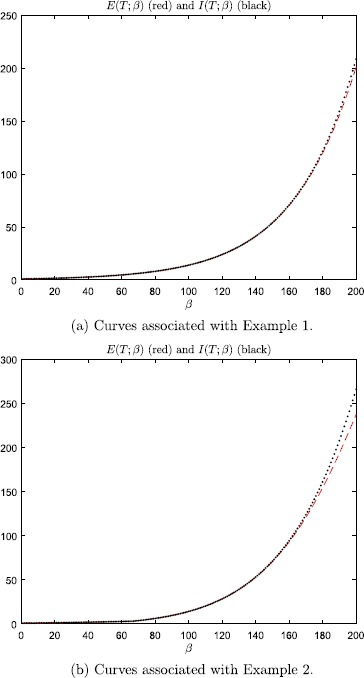
Error-amplification ratio $E(T;\beta)$, *red dashed lines*, as a function of the steepness parameter *β*. *The black dotted curves* are the graphs of $I(T;\beta)$. These plots were generated with $T=0.06$

In our numerical experiments, $\| \mathbf{u}_{0} - \tilde{\mathbf {u}}_{0} \| _{\infty} = 10^{-5}$ and $\beta_{\max}=200$. That is, $\| \mathbf{u}_{0} - \tilde{\mathbf{u}}_{0} \|_{\infty} \ll\beta_{\max}^{-1}$ and (14) will hold with some $\alpha\geq1$ during a short time interval $[0,\hat{T}]$. It is virtually impossible to distinguish between the curves of $E(T;\beta)$ and $I(T;\beta)$, $\beta=1,2, \ldots , 200$, when $T=10^{-4}$ (curves not presented). Figure 8 illustrates that $I(T;\beta)$ also yields a reasonable approximation of $E(T;\beta)$ for $T=0.06$.

We conclude that, during time intervals in which (14) holds, the linearized equations (9)–(10) yield a fair approximation of the point-neuron model (1)–(2). Hence, the analysis presented in this section, which provided an error-amplification ratio of order $O(e^{\beta})$ for (9)–(10), explains our numerical results. More precisely, even though the error is bounded by $2 \beta_{\max}^{-1-\alpha}$ during such time intervals, see (15), the error-amplification ratio can approximately be of order $O(e^{\beta})$. This implies that minor perturbations, e.g. round-off errors, can corrupt computations. For example, in Fig. 4 an initial perturbation of size 10^−5^ is increased to an error of approximately $0.04=4~\%$.

#### Remark

Assume that the $\| \cdot\|_{\infty}$-norm of the source term $\mathbf {q}(t)$ is bounded. Then, since $0< S_{\beta}[x] < 1$ for all $x \in \mathbb {R}$, it follows from (1) that both $\| \mathbf{u}'(t) \|_{\infty}$ and $\| \tilde{\mathbf{u}}'(t) \| _{\infty}$ are bounded independently of the size of the steepness parameter *β*, at least when $\mathbf{u}(t)\approx u_{\theta}$ and $\tilde{\mathbf{u}}(t) \approx u_{\theta}$. Consequently, also the difference $\| \mathbf{u}(T) - \tilde{\mathbf{u}}(T) \|_{\infty}$ is bounded independently of $\beta> 0$. Our results therefore might appear to be somewhat counter-intuitive: But note that we have only argued that the error-amplification ratio (5) may, approximately, be of order $O(e^{\beta})$. If *β* is large, this can cause severe numerical challenges.

We would also like to comment that standard theory for general dynamical systems

$$\begin{aligned} \mathbf{z}'(t) =&\mathbf{F}\bigl(t,\mathbf{z}(t)\bigr),\quad t \in(0,T], \\ \mathbf{z}(0) =& \mathbf{z}_{0}, \end{aligned}$$

relies on the size of $\| \mathbf{F}' \|$, which for the point-neuron model (1)–(2) is of order $O(\beta)$. Also, $\mathbf{F}(t,\mathbf{z}) = -\mathbf{z}+ \omega S_{\beta }[\mathbf {z}-\mathbf{u}_{\theta}] + \mathbf{q}(t)$ is not Lipschitz continuous with respect to **z** when $\beta= \infty$, which the Picard–Lindelöf theorem [9, 10] requires. (**F** is not even continuous when $\beta=\infty$.)

The maximum error bound (15), valid when $\mathbf {u}-\mathbf{u}_{\theta}$ and $\tilde{\mathbf{u}}-\mathbf {u}_{\theta}$ satisfy (14), suggests that setting $\beta= \infty$ might provide a solution to the issues discussed above. Unfortunately, as will be explained in the next section, this is not the case.

## Ill Posed

We will now show that (1)–(2) can become truly ill posed, if a Heaviside firing rate function is employed. More specifically, the *initial-condition*-to-*solution* map, in finite time, can be discontinuous.

Consider the case $N=1$, $\tau=1$ and no source term:

22 $$ \begin{aligned} &v'(t)=-v(t) + \omega H \bigl[v(t)-u_{\theta}\bigr], \\ &v(0)=u_{0}. \end{aligned} $$

If, for $0 < \epsilon\ll1$,

$$u_{0} = u_{\theta}+\epsilon> u_{\theta} \quad\mbox{and}\quad \widetilde{u}_{0}=u_{\theta}-\epsilon< u_{\theta}, $$

then

$$\begin{aligned} v(t) =& \omega+(u_{\theta}+\epsilon-\omega)e^{-t}, \\ \widetilde{v}(t) =& (u_{\theta}-\epsilon) e^{-t}, \end{aligned}$$

provided that $\omega> u_{\theta}$. Consequently,

$$\bigl|R_{\infty}(u_{0})-R_{\infty}(\widetilde{u}_{0})\bigr| = \bigl| v(T)-\widetilde{v}(T)\bigr| =\bigl|\omega\bigl(1-e^{-T}\bigr) + 2 \epsilon e^{-T}\bigr| > \omega\bigl(1-e^{-T}\bigr), $$

where $R_{\infty}$ denotes the *initial-condition*-to-*solution* map (3). We conclude that, no matter how close $u_{0}$ and $\widetilde{u}_{0}$ are, the difference $v(T)-\widetilde{v}(T)$ between the corresponding solutions will not become small. Hence, $R_{\infty}$ is discontinuous. It follows that the initial value problem, with a Heaviside firing rate function, is ill posed, in finite time—at least in the sense of Hadamard. Also note that, unless $\omega=2 u_{\theta}$, $u_{\theta}$ is not a stationary solution of (22), i.e. not an unstable equilibrium.

The error-amplification ratio for this ill-posed problem becomes infinite when $\epsilon\rightarrow0$:

$$\begin{aligned} \frac{| v(T)-\widetilde{v}(T)|}{|u_{0} - \widetilde{u}_{0}|} =& \frac {|R_{\infty}(u_{0})-R_{\infty}(\widetilde{u}_{0})|}{|u_{0} - \widetilde {u}_{0}|} \\ =& \frac{|\omega(1-e^{-T}) + 2 \epsilon e^{-T}|}{2 \epsilon} \\ >& \frac{\omega(1-e^{-T})}{2 \epsilon} \rightarrow\infty \end{aligned}$$

as $\epsilon\rightarrow0$, for any $T>0$.

One may consider this issue from a more pragmatic point of view. Let $v_{\Delta t}$ denote a numerical approximation of *v*. If a Heaviside firing rate function is employed, then $H(v_{\Delta t}-u_{\theta})$ must be evaluated in some line of the simulation software. This is an unstable procedure because *H* has a jump discontinuity at 0, and round-off errors of any size can corrupt computations.

In contrast to this, provided that $\beta< \infty$,

23 $$\begin{aligned} \bigl\| \mathbf{u}(T)-\tilde{\mathbf{u}}(T)\bigr\| _{\infty}\leq\| \mathbf{u}_{0} -\tilde{\mathbf{u}}_{0}\|_{\infty}\cdot \exp\bigl[(A+\beta B)T\bigr],& \end{aligned}$$

see the analysis of the model (1)–(2) presented in Appendix A. Here, $\tilde{\mathbf{u}}_{0}$ is any perturbation of the initial condition $\mathbf{u}_{0}$, and *A* and *B* are positive constants depending on the matrices ***τ*** and ***ω***, but not on *β*. This inequality shows that the *initial-condition*-to-*solution* map $R_{\beta}$, $\beta< \infty$, also is continuous at unstable equilibria.

## Conclusions and Discussion

Since $R_{\infty}$ can become discontinuous, it is virtually impossible to guarantee the accurate numerical solution of point-neuron models which employ a Heaviside firing rate function: Any round-off errors can potentially corrupt simulations. Alternatively, one may stop the simulation as soon as the solution hits the jump discontinuity, i.e. the threshold value for firing.

We have also observed that models with a steep, but smooth, firing rate function can amplify errors to an extreme degree, which is typical for “almost ill-posed” problems. Consequently, reliable simulations can only be obtained if proper error-control schemes are invoked. How to design effective error-control methods, for models with a large steepness parameter *β*, is, as far as the authors know, still an open problem. Nevertheless, it seems plausible that suitable adaptive numerical schemes, where the time steps become smaller when the solution reaches regions in the vicinity of the threshold value for firing, might be capable of handling the numerical error amplification.

Let

$$F_{\beta; t_{1},t_{2}}: \mathbf{u}(t_{1}) \rightarrow\mathbf{u}(t_{2}), \quad t_{2} > t_{1} \geq0, $$

be the operator which maps the solution of the point-neuron model (1)–(2) from time $t_{1}$ to time $t_{2}$. Note that the action of $F_{\beta;t_{1},t_{2}}$ can be determined by solving the point-neuron model with $\mathbf{u}(t_{1})$ as initial condition. Therefore, from the argument presented above, it follows that the error amplification ratio associated with $F_{\beta; t_{1},t_{2}}$ may be large, provided that $\beta\gg1$. We conclude that the issues pointed out in this study cannot necessarily be avoided by using an initial condition which is far from the threshold value $\mathbf{u}_{\theta}$ for firing. In fact, it seems that one must prove that $\mathbf{u}(t)$ never gets close to $\mathbf {u}_{\theta}$ for $t>0$—a herculean task, if correct.

From a modeling perspective one might wonder: Should a voltage-based model of cortex be ill posed or “almost ill posed”? If so, then models employing a Heaviside firing rate function cannot be robustly solved with finite precision arithmetic and regularized approximations are numerically challenging [4, 5].

We fear that similar unfortunate properties, to those discussed in this paper, might be valid for models which can be written in the form

$$\begin{aligned} \mathbf{z}'(t) =&\mathbf{F}_{\beta}\bigl(t,\mathbf{z}(t) \bigr), \quad t \in(0,T], \\ \mathbf{z}(0) =& \mathbf{z}_{0}, \end{aligned}$$

where $\| \mathbf{F}'_{\beta} \| \rightarrow\infty$ when $\beta \rightarrow\infty$. This can, e.g., be the case for a number of models in use in computational neuroscience and gene regulatory networks.

An easy solution to the issues raised in this paper, is to avoid steep firing rate functions. If *β* is fairly small, then standard ODE theory [9, 10] and textbook material about their numerical treatment can be used, provided that the source term $\mathbf {q}(t)$ is continuous. Nevertheless, steep sigmoid functions are popular in computational neuroscience.

## Appendix A: Continuous Dependence on the Initial Condition

We will prove the stability estimate (23), which holds if $\beta< \infty$. Let **u** and $\tilde{\mathbf{u}}$ denote the solutions of (1) corresponding to the initial conditions $\mathbf{u}_{0}$ and $\tilde{\mathbf{u}}_{0}$, respectively. For the purpose of detailing the derivation of this estimate, we work with the components of these vector functions, i.e.,

$$\begin{aligned} \mathbf{u}(t) =&\left ( \textstyle\begin{array}{@{}c@{}} u_{1}(t) \\ u_{2}(t)\\ \vdots\\ u_{N}(t) \end{array}\displaystyle \right ),\qquad \tilde{\mathbf{u}}(t)=\left ( \textstyle\begin{array}{@{}c@{}} \tilde{u}_{1}(t) \\ \tilde{u}_{2}(t)\\ \vdots\\ \tilde{u}_{N}(t) \end{array}\displaystyle \right ), \\ \mathbf{u}_{0} =&\left ( \textstyle\begin{array}{@{}c@{}} u_{1,0} \\ u_{2,0}\\ \vdots\\ u_{N,0} \end{array}\displaystyle \right ), \qquad\tilde{\mathbf{u}}_{0}=\left ( \textstyle\begin{array}{@{}c@{}} \tilde{u}_{1,0} \\ \tilde{u}_{2,0}\\ \vdots\\ \tilde{u}_{N,0} \end{array}\displaystyle \right ). \end{aligned}$$

Let us also assume that the diagonal entries $\{ \tau_{i} \}$, of the diagonal matrix ***τ***, are positive, and let $\{ \omega_{ij} \}$ denote the entries of ***ω***.

We first observe that the component functions $u_{i}$ and $\tilde {u}_{i}$ satisfy the fixed point problems

$$\begin{aligned} u_{i}(T) =&u_{i,0}+\tau_{i}^{-1} \int_{0}^{T}\Biggl\{ -u_{i}(t)+\sum _{j=1}^{N}\omega_{ij}S_{\beta} \bigl[u_{j}(t)-u_{j,\theta}\bigr]+q_{i}(t)\Biggr\} \,dt,\\ \tilde{u}_{i}(T) =&\tilde{u}_{i,0}+\tau_{i}^{-1} \int_{0}^{T}\Biggl\{ -\tilde{u}_{i}(t)+ \sum_{j=1}^{N}\omega_{ij}S_{\beta} \bigl[\tilde{u}_{j}(t)-u_{j,\theta}\bigr]+q_{i}(t) \Biggr\} \,dt, \end{aligned}$$

from which we find, by using the triangle inequality, that

$$\begin{aligned} \bigl|u_{i}(T)-\tilde{u}_{i}(T)\bigr| \leq& |u_{i,0}- \tilde{u}_{i,0}| + \tau_{i}^{-1} \int_{0}^{T}\bigl|u_{i}(t)- \tilde{u}_{i}(t)\bigr|\,dt \\ &{}+ \tau_{i}^{-1}\sum_{j=1}^{N}| \omega_{ij}| \int_{0}^{T} \bigl|S_{\beta} \bigl[u_{j}(t)-u_{j,\theta}\bigr]-S_{\beta}\bigl[\tilde {u}_{j}(t)-u_{j,\theta}\bigr]\bigr|\,dt. \end{aligned}$$

Then, by exploiting the fact that

$$\bigl|S_{\beta}(x)-S_{\beta}(y)\bigr|\leq\frac{1}{2}\beta|x-y| $$

for all $x,y\in \mathbb {R}$, we get

$$\begin{aligned} \bigl|u_{i}(T)-\tilde{u}_{i}(T)\bigr| \leq& |u_{i,0}- \tilde{u}_{i,0}| \\ &{} + \tau_{i}^{-1} \int_{0}^{T}\bigl|u_{i}(t)- \tilde{u}_{i}(t)\bigr|\,dt \\ &{} + \tau_{i}^{-1} \frac{1}{2}\beta\sum _{j=1}^{N}|\omega_{ij}| \int_{0}^{T}\bigl|u_{j}(t)- \tilde{u}_{j}(t)\bigr|\,dt \\ \leq& |u_{i,0}-\tilde{u}_{i,0}| \\ &{} + \tau_{i}^{-1} \Biggl( 1+ \frac{1}{2} \beta\sum_{j=1}^{N}|\omega_{ij}| \Biggr) \int_{0}^{T}\bigl\| \mathbf{u}(t)-\tilde{\mathbf{u}}(t) \bigr\| _{\infty}\,dt, \end{aligned}$$

where we in the final step have used the definition of the supremum norm (4). Thus, we arrive at the inequality

$$\begin{aligned} \bigl\| \mathbf{u}(T)-\tilde{\mathbf{u}}(T)\bigr\| _{\infty}\leq {}&\|\mathbf {u}_{0}-\tilde{\mathbf{u}}_{0}\|_{\infty}\\ &{}+\max _{1\leq i\leq N} \Biggl[ \tau_{i}^{-1} \Biggl( 1+ \frac{1}{2}\beta\sum_{j=1}^{N}| \omega_{ij}| \Biggr) \Biggr] \int_{0}^{T}\bigl\| \mathbf{u}(t)-\tilde{\mathbf{u}}(t) \bigr\| _{\infty}\,dt. \end{aligned}$$

The stability estimate (23), with

$$A=\max_{1\leq i\leq N}\bigl({\tau_{i}}^{-1}\bigr),\qquad B=\frac{1}{2}\max_{1\leq i\leq N} \Biggl( {\tau_{i}}^{-1} \sum_{j=1}^{N}|\omega_{ij}| \Biggr), $$

then follows from Grönwall’s lemma.

## Appendix B: Continuous Dependence on the Steepness Parameter

For the sake of completeness, we also show that the solution of the initial value problem (1)–(2) depends continuously on the steepness parameter *β*. Let *β* and *β̂* be steepness parameters for the firing rate function. We fix the initial condition and the connectivity parameters of (1)–(2). The solutions corresponding to *β* and *β̂* are denoted by **u** and $\hat{\mathbf{u}}$, respectively. We readily obtain

$$\begin{aligned} u_{i}(T) =&u_{i,0}+\tau_{i}^{-1} \int_{0}^{T}\Biggl\{ -u_{i}(t)+\sum _{j=1}^{N}\omega_{ij}S_{\beta} \bigl[u_{j}(t)-u_{j,\theta}\bigr]+q_{i}(t)\Biggr\} \,dt,\\ \hat{u}_{i}(T) =&u_{i,0}+\tau_{i}^{-1} \int_{0}^{T}\Biggl\{ -\hat{u}_{i}(t)+ \sum_{j=1}^{N}\omega_{ij}S_{\hat{\beta}} \bigl[\hat{u}_{j}(t)-u_{j,\theta}\bigr]+q_{i}(t)\Biggr\} \,dt, \end{aligned}$$

for the component functions $u_{i}$ and $\hat{u}_{i}$ of **u** and $\hat{\mathbf{u}}$, respectively. We now make use of the property

$$ \bigl|S_{\beta}[u_{j}-u_{j,\theta}]-S_{\hat{\beta}}[ \hat{u}_{j}-u_{j,\theta}]\bigr| \leq\frac{1}{2}\bigl[ \beta|u_{j}-\hat{u}_{j}|+|\beta-\hat{\beta }|\bigl(|u_{j,\theta}|+|\hat{u}_{j}|\bigr)\bigr], $$

for the firing rate function, and we get the chain of inequalities

24 $$\begin{aligned} \bigl|u_{i}(T)-\hat{u}_{i}(T)\bigr| \leq&\tau_{i}^{-1} \int_{0}^{T}\bigl|u_{i}(t)- \hat{u}_{i}(t)\bigr|\,dt \\ &{}+\tau_{i}^{-1}\sum_{j=1}^{N}| \omega_{ij}| \int_{0}^{T}\bigl\{ \bigl|S_{\beta} \bigl[u_{j}(t)-u_{j,\theta}\bigr]-S_{\hat{\beta}}\bigl[\hat {u}_{j}(t)-u_{j,\theta}\bigr]\bigr|\bigr\} \,dt \\ \leq&\tau_{i}^{-1} \int_{0}^{T}\bigl|u_{i}(t)- \hat{u}_{i}(t)\bigr|\,dt \\ &{}+\frac {1}{2}\beta\tau_{i}^{-1} \sum_{j=1}^{N}|\omega_{ij}| \int_{0}^{T}\bigl|u_{j}(t)- \hat{u}_{j}(t)\bigr|\,dt \\ &{}+\frac{1}{2}|\beta-\hat{\beta}|\tau_{i}^{-1}\sum _{j=1}^{N}|\omega_{ij}| \biggl( |u_{j,\theta}|T+ \int_{0}^{T}\bigl|\hat{u}_{j}(t)\bigr|\,dt \biggr). \end{aligned}$$

Since $0\leq S(x)\leq1$, we find the bound

$$\begin{aligned} \bigl|\hat{u}_{j}(s)\bigr| \leq& C_{j}(s),\quad s \in[0,T], \\ C_{i}(s) \equiv&\sum_{j=1}^{N}| \omega_{ij}| +\biggl| \int_{0}^{s}\alpha_{i}(s-t)q_{i}(t)\,dt\biggr| + \Biggl( |u_{i,0}|-\sum_{j=1}^{N}| \omega_{ij}| \Biggr) \tau_{i}\alpha_{i}(s)\\ =&\sum_{j=1}^{N}| \omega_{ij}| \bigl(1-\tau_{i}\alpha_{i}(s)\bigr) +\biggl| \int_{0}^{s}\alpha_{i}(s-t)q_{i}(t)\,dt\biggr| +|u_{i,0}| \tau_{i}\alpha_{i}(s), \end{aligned}$$

uniformly in *β*, for the solutions of (1)–(2). Here,

$$ \alpha_{i}(t)=\frac{1}{\tau_{i}}e^{-t/\tau_{i}}, $$

and it follows that the integrals $\int_{0}^{T}|\hat {u}_{j}(t)|\,dt$ in (24) can be bounded by *β*-independent constants. Thus, we end up with the bounding inequality

$$\bigl\| \mathbf{u}(T)-\hat{\mathbf{u}}(T)\bigr\| _{\infty}\leq C(T) |\beta-\hat{ \beta}|+(A+\beta B) \int_{0}^{T}\bigl\| \mathbf{u}(t)-\hat{\mathbf{u}}(t) \bigr\| _{\infty}\,dt, $$

where *A* and *B* are as defined in Appendix A and

$$ C(T)=\frac{1}{2}\sup_{t^{*} \in[0,T]} \max_{1\leq i\leq N} \Biggl\{ \tau_{i}^{-1} \sum_{j=1}^{N}| \omega_{ij}| \biggl( |u_{j,\theta}|t^{*} + \int_{0}^{t^{*}}C_{j}(t)\,dt \biggr) \Biggr\} . $$

Grönwall’s lemma yields the stability estimate

25 $$ \bigl\| \mathbf{u}(T)-\hat{\mathbf{u}}(T)\bigr\| _{\infty}\leq \underbrace{C(T)|\beta- \hat{\beta}|\cdot\exp\bigl[(A+\beta B)T\bigr]}_{q(T)}, $$

which proves that the solution of (1)–(2) depends continuously on the steepness parameter $\beta< \infty$ of the firing rate function.

Since $q(T)$ is increasing, it is possible to extract further information from this argument. More specifically, let

26 $$ U= \Bigl\{ \mathbf{v}(t) \bigm| \mathbf{v}(t) \in \mathbb {R}^{N}, t \in[0,T] \mbox{ and } \sup_{t \in[0,T] } \bigl\| \mathbf{v}(t) \bigr\| _{\infty} < \infty\Bigr\} , $$

with norm

27 $$ \| \mathbf{v} \|_{U} = \sup_{t \in[0,T] } \bigl\| \mathbf{v}(t) \bigr\| _{\infty}. $$

Then we can conclude that the mapping

$$\begin{aligned} & \mathbb {R}_{+} \rightarrow U,\\ & \beta\rightarrow u(t; \beta),\quad t \in[0,T], \end{aligned}$$

is continuous.

## Appendix C: Continuous Dependence on the Initial Condition and the Steepness Parameter

We finally prove that the solution $\mathbf{u}=\mathbf{u}(t;\mathbf {u}_{0},\beta)$ of (1)–(2) depends continuously on $(\mathbf{u}_{0},\beta)$. The proof of this fact proceeds as follows: By exploiting the triangle inequality and the stability estimates (23) and (25), we find that

$$\begin{aligned} &\bigl\| \mathbf{u}(T;\tilde{\mathbf{u}}_{0},\hat{\beta})-\mathbf{u}(T; \mathbf{u}_{0},\beta)\bigr\| _{\infty}\\ &\quad\leq\bigl\| \mathbf{u}(T;\tilde{\mathbf{u}}_{0},\hat{\beta })-\mathbf {u}(T;\mathbf{u}_{0},\hat{\beta})\bigr\| _{\infty}+ \bigl\| \mathbf{u}(T; \mathbf{u}_{0},\hat{\beta})-\mathbf{u}(T;\mathbf{u}_{0}, \beta)\bigr\| _{\infty}\\ &\quad\leq\underbrace{\bigl\{ \|\tilde{\mathbf{u}}_{0}- \mathbf{u}_{0} \|_{\infty }+C(T)|\hat{\beta}-\beta|\bigr\} \cdot \exp\bigl[(A+\beta B)T\bigr]}_{g(T)} \end{aligned}$$

and we are done. Here $\mathbf{u}_{0}$ and $\tilde{\mathbf{u}}_{0}$ denote two initial conditions of (1), while *β̂* and *β* are two steepness parameters.

The function $g(T)$ is increasing, and we conclude that the mapping

$$\begin{aligned} & \mathbb {R}^{N} \times \mathbb {R}_{+} \rightarrow U,\\ & (\mathbf{u}_{0},\beta) \rightarrow\mathbf{u}(t; \mathbf{u}_{0},\beta),\quad t \in[0,T], \end{aligned}$$

where *U* is defined in (26)–(27), is continuous.
